# Effect of erector spinae plane block with different doses of dexmedetomidine as adjuvant for ropivacaine on the postoperative quality of recovery after video-assisted thoracoscopic lobectomy surgery: a randomized controlled trial

**DOI:** 10.1186/s12871-023-02231-9

**Published:** 2023-08-07

**Authors:** Yanxia Guo, Jingting Wang, Pingping Jiang, Dan Wang, Wenxi Fan, Xiaolin Yang

**Affiliations:** Department of Anaesthesia, Affiliated Hospital of North Sichuan Medical College, Nanchong, 637000 Sichuan China

**Keywords:** Erector spinae plane block, Dexmedetomidine, Ropivacaine, Postperative, Quality of recovery, Video-assisted thoracic surgery, Ultrasonography

## Abstract

**Background:**

Erector spinae plane block (ESPB) improves postoperative analgesia and significantly enhances the quality of recovery (QoR) after video-assisted thoracoscopic lobectomy surgery (VATLS). However, it is not known whether the use of dexmedetomidine (Dex) as an adjunct for ropivacaine to ESPB affects the QoR after VATLS. The purpose of this study was to explore the effects of different Dex dosages as an adjunct for ropivacaine in combination with ultrasound-guided ESPB on the quality of postoperative recovery in patients with VATLS.

**Methods:**

In this single-center, double-blind, randomized study, 120 patients between the ages of 18 and 65 who were scheduled for VATLS from december 2021 and october 2022 in our hospital under general anesthesia were randomly divided into three groups: ultrasound-guided ESPB with 30 mL of 0.5% ropivacaine (Group R), ultrasound-guided ESPB 0.5% ropivacaine plus 0.5 µg/kg Dex (Group RD1), and ultrasound-guided ESPB 0.5% ropivacaine plus 1.0 µg/kg Dex (Group RD2), ultrasound-guided ESPB was administrated at the T5 vertebral level before surgery. The primary outcome was the QoR-15 score 24 h after the surgery. The secondary outcomes included the QoR-15 scores at 12 h, 48 h, and 72 h after the operation, visual analogue scale (VAS) scores at 8 h, 12 h, 24 h, and 48 h after surgery, cumulative flurbiprofen consumption, postoperative nausea and vomiting (PONV), postoperative bradycardia, and hypotension.

**Results:**

The QoR-15 scores were higher in group RD2 than the R and RD1 groups on postoperative day 1 (P < 0.05), in addition, no significant difference was found in the QoR-15 scores between groups R and RD1 on postoperative day 1. The VAS scores were significantly lower in group RD2 than in groups RD1 and group R 12–24 h after surgery (P < 0.05). No significant differences were observed in the QoR-15 and VAS scores at 48 and 72 h after surgery between the three groups. The cumulative flurbiprofen consumption was markedly reduced during the 72 h after surgery in the RD2 group (P < 0.05). The incidence of postoperative nausea and vomiting was lower in the RD2 group (P < 0.05).

**Conclusions:**

The combination of 1 µg/kg dexmedetomidine as an adjunct with 0.5% ropivacaine 30 ml for erector spinae plane block significantly improved the postoperative quality of recovery and provided better postoperative analgesia on postoperative day 1 in patients undergoing Video-assisted thoracoscopic lobectomy surgery. However, dexmedetomidine (1 µg/kg) as an adjunct for ropivacaine combined with erector spinae plane block did not enhance the postoperative quality of recovery at 48 and 72 h postoperatively.

**Trial registry number:**

The number of this clinical trial registry is ChiCTR2100053230, date of registration: 16/11/ 2021)

## Introduction

The rapid development of minimally invasive technology has hastened postoperative recovery. The rapid recovery of normal daily activities is the primary goal of perioperative anesthetic management for patients. Video-assisted thoracoscopic lobectomy surgery (VATLS) can effectively reduce the postoperative complication rate and promote early recovery after thoracic surgery in comparison with traditional open thoracotomy [1, 2]. However, for many anesthesiologists and thoracic surgeons, postoperative pain management, especially early postoperative pain, remains a source of concern [3]. Postoperative pain after VATLS might result in several pulmonary complications, such as atelectasis and hypercapnia, as well as lung infection, which can affect the quality of recovery(QoR) [4, 5].

Regional anesthesia is now considered a core component of multimodal analgesia after surgery. However, the optimal analgesic technique for video-assisted thoracoscopic lobectomy surgery is not well defined. Erector spinae plane block (ESPB) has been applied due to its ease of use, safety, and efficacy. ESPB has been found to substantially improve postoperative analgesia and enhance the QoR after surgery [6–8]. Recently, several clinical studies have reported that dexmedetomidine (Dex) could be potentially utilized as an adjuvant to ESPB with ropivacaine to prolong the duration of postoperative analgesia in many types of surgeries, including thoracoscopic surgery [9–11]. However, it remains unclear whether Dex as an adjunct for ropivacaine to ESPB can significantly improve the quality of postoperative recovery after VATLS. Thus, the main objective of the present study was to explore the effects of different dosages of Dex as an adjunct for ropivacaine combined with ESPB on the quality of postoperative recovery in patients following VATLS.

## Materials & methods

### Study design and randomization

The study was approved by the Institutional Ethics Committee (Approval No. 2202ER384-1) and was registered in the Center of Chinese Clinical Trials Registry at http://www.chictr.org.cn (ChiCTR2100053230). Written informed consent was obtained from all participants before enrollment. This study was performed in accordance with the principles of the Declaration of Helsinki. All patients with an American society of anesthesiologists (ASA) physical status I-II, aged 18–65 years, and scheduled for VATLS were recruited to our study which was conducted between December 2021 and October 2022. The exclusion criteria included a patient’s refusal to sign informed consent, body mass index < 18 or > 30 kg/m^2^, infection at the puncture site, known coagulation disorders, and patients who had a history of chest surgery and chronic pain or use of opioid and Dex, allergy to local anesthetics or Dex, or suffered from mental disease and were unable to cooperate, central nervous system disorders that could prevent completion of the QoR-15 questionnaire.

All the patients were randomly divided into three groups using a computer-generated random-number list and a 1:1:1 allocation ratio. Group R received 30 mL of 0.5% ropivacaine. Group RD1 received 0.5% ropivacaine plus 0.5 µg/kg Dex and Group RD2 received 0.5% ropivacaine with 1 µg/kg Dex. An independent anaesthesia nurse who was not involved in the study prepared the study medication on the day of surgery in a randomized order hidden in an opaque envelope, which were opened after the patients had entered the operating theater. To ensure blinding, the volume of the study medication was standardized at 30 mL for all groups and injected by ultrasound-guided ESPB block. All the other investigators and all the patients were blinded to the group allocation throughout the entire perioperative period. The patients were also taught how to complete the global QoR-15 questionnaire and the VAS score before the surgery.

### Standard general anesthesia

All the patients were routinely fasted and did not take any preoperative medication. Noninvasive blood pressure, heart rate, electrocardiography, pulse oxygen saturation, and the Bispectral index (BIS) were monitored after arrival in the operating theater. The general anesthetic induction was conducted with propofol 2 mg/kg, sufentanil 0.4 µg/kg, and cisatracurium 0.15 mg/kg and double-lumen tracheal intubation was initiated to accomplish lung isolation when the patient had lost consciousness and the BIS index value had decreased to below 40. After intubation, the mechanical ventilation was set with 100% oxygen at a tidal volume of 6 to 8 ml/kg, an inspiratory-to-expiratory ratio of 1:2, and a respiratory frequency of 12–16 breaths/min. The ventilation was switched to one-lung ventilation at the time of skin incision. A normal end-tidal carbon dioxide (CO_2_) tension (35 to 45 mmHg) was maintained by adjusting the respiratory frequency and the tidal volume intraoperatively. The general anesthesia was maintained with inhalation of sevoflurane 2–4% and continuous infusion of remifentanil at 0.05-2 µg/kg/min to maintain the BIS value between 40 and 60. Cisatracurium were used as required.

### Ultrasound-guided erector spinae plane block

After the induction of general anesthesia, the patients were placed in a standard lateral position to apply ESPB under aseptic conditions. All the blocks were performed by a senior anesthesiologist with extensive experience in ultrasound-guided nerve blocks. A high-frequency linear ultrasound probe was first placed longitudinally 3 cm from the midline of the T5 level of the spine. An in-plane technique was then used after imaging the trapezius, rhomboid major, erector spinae, and T5 transverse processes. The ESPB procedure entailed injecting local anesthetics deep into the erector spinae muscles and between the transverse processes in the fascial plane. Thereafter, a 22-G, 80-mm needle was inserted in a caudal to the cephalad orientation under ultrasound guidance after standard skin disinfection. The correct position of the needle tip was confirmed by injecting 2 mL of 0.9% normal saline that separated the transverse process from the erector spinae muscle. After determination of the correct needle tip position, the anesthesiologist slowly injected 30 ml of 0.5% ropivacaine, with or without administering the different doses of Dex.

### Postoperative analgesia protocol

Propofol and remifentanil were discontinued at the end of the surgery, the double-lumen tracheal tubes were extubated when they met the criteria for tracheal tube removal, and then the patients were transferred to the post-anesthesia care unit (PACU). A patient-controlled intravenous anesthesia (PCIA) pump containing sufentanil 1 µg/ml and butorphanol 50 µg/ml was provided for all the patients at the end of the procedure. The PCIA pump was programmed to deliver a bolus amount of 2 mL and a background dose of 2.5 mL/h, as well as having a lockout period of 15 min. However, if the VAS score exceeded 3, a bolus injection of 2 µg sufentanil and 100 µg butorphanol was given through the PCIA to alleviate the pain with a 15-minute lockout period. However, if the VAS score remained above 3 after using the PCIA, the patients received flurbinprofen 8 mg as a rescue analgesic. The PCIA devices were removed 48 h after the surgery. Tropisetron 5 mg was used to treat postoperative nausea and vomiting (PONV).

### Outcome measures

The primary outcome was the QoR-15 score at 24 h postoperatively, which was evaluated using the QoR-15 questionnaire. Higher QoR-15 scores generally indicate better quality of patient recovery following surgery. The QoR-15 questionnaire is a multimodal, validated patient-centered quality evaluation tool used for the comprehensive assessment of the quality of postoperative recovery and patient satisfaction [12]. Which covers five clinical dimensions, including physical comfort (five items), physical independence (two items), psychological support (two items), and pain (two items), emotional state(four items) [13].

The secondary outcomes were the QoR-15 scores at 12 h, 48 h, and 72 h. The VAS scores were monitored at 4 h, 8 h, 12 h, 24 h, 48 h, and 72 h postoperatively, and the total times that PCIA was pressed, the time of first request for flurbiprofen, and cumulative flurbiprofen consumption on the postoperative days (POD)1, POD2, and POD3. Various perioperative side effects, such as heart arrhythmia, hypotension, nausea and vomiting, and dizziness were also measured.

### Sample size and statistical analyses

The sample size was determined based on a pilot research that revealed the 24 h postoperative QoR-15 scores for the groups R, RD1, and RD2 were 106.3 ± 11.3, 110.6 ± 12.4, 116.7 ± 16.8, respectively. The minimum desired clinical difference between the groups was an 8-point difference for the QoR-15 score [14], and 38 patients per group were needed to obtain a significance level of 0.05 with an 90% power. Accounting for dropouts, we enrolled 120 participants in this study.

Statistical analysis was performed using SPSS 25.0 software (SPSS 25, Chicago, IL, USA). The Kolmogorov-Smirnov test was employed to evaluate the normality of the data distribution. The continuous variables were presented as mean (SD) or median (interquartile range), as appropriate. Normally distributed continuous data were analyzed with one-way analysis of variance tests and non-normally distributed continuous data were analyzed using the non-parametric Kruskal-Wallis H test. The categorical variables were presented as numbers (percentages) and analyzed using the *X*^2^ test. The Bonferroni^,^s post-hoc test was used following inter-group comparisons. P values < 0.05 were considered statistically significant.

## Results

**Fig. 1:**
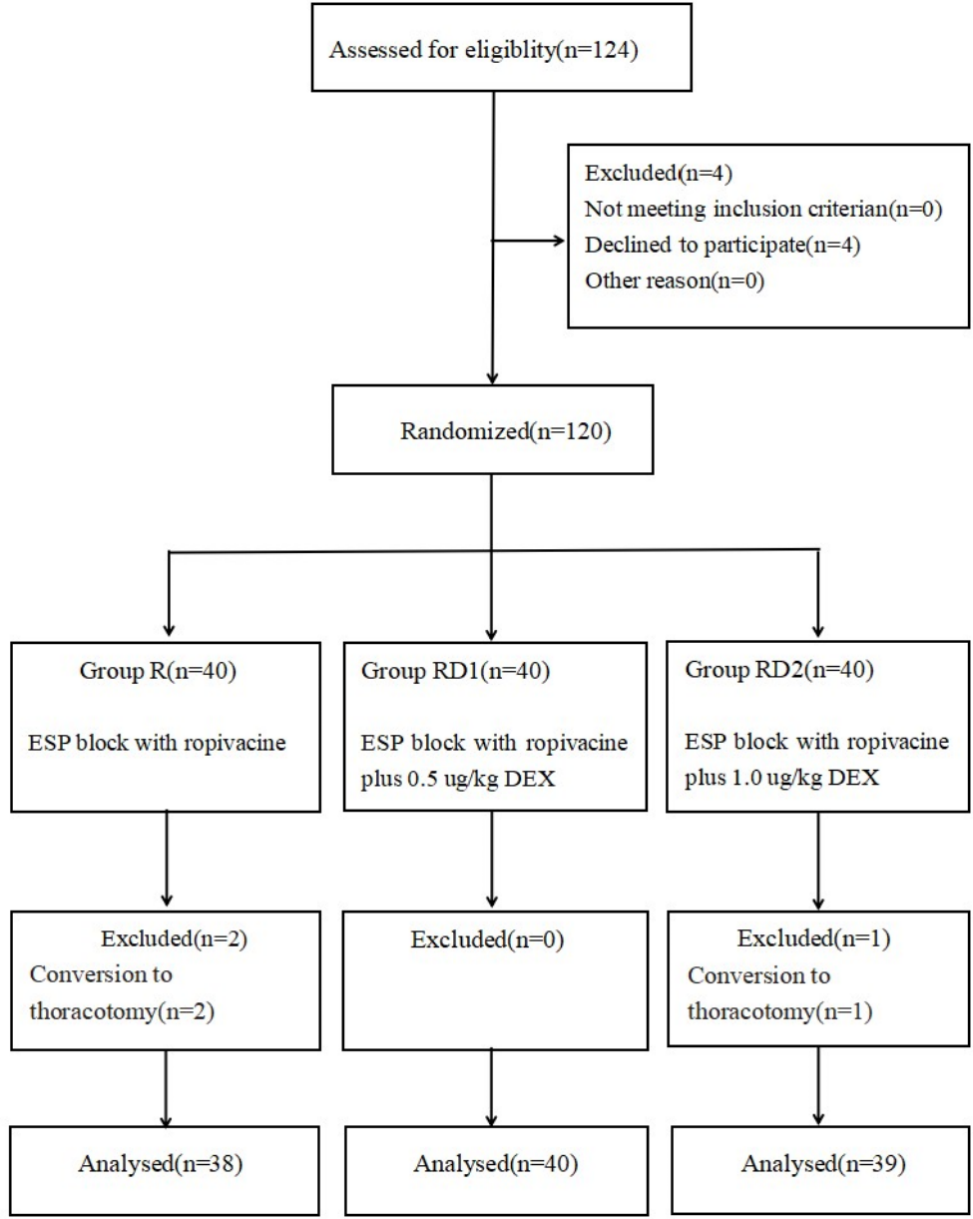
CONSORT flow diagram. CONSORT, Consolidated Standards of Reporting Trials

A total of 120 patients were enrolled in the study from December 2021 to October 2022. However, two patients in group R and one patient in group DR2 were converted to thoracotomy during surgery, all these patients were excluded from the study. Consequently, 117 patients ( 38, 40, 39 patients in group R, DR1, DR2, respectively) completed the study (Fig. 1). No significant differences were found in the demographic data of patients among the three groups (Table 1).

**Table 1:** Demographic data and intraoperative data of the enrolled patients

	Group R	Group RD1	Group RD2
Age(yr)	52.9 ± 8.7	53.6 ± 7.7	50.9 ± 10.6
Height(cm)	163.5 ± 5.8	162.7 ± 6.2	164.7 ± 5.3
Weight(kg)	63.0 ± 6.3	58.5 ± 7.1	62.0 ± 8.1
BMI(kg/m^2^)	23.7 ± 1.8	23.1 ± 2.7	23.0 ± 2.5
Gender(n, M/F)	18/20	19/21	21/18
ASAclass(I-II)	16/22	20/20	17/22
Surgical side(right/left)	21/17	18/22	20/19
Operation time(min)	119.5 ± 10.6	123.8 ± 15.3	122.3 ± 16.7
Consumption of sufentanil (µg)	55.6 ± 5.3	53.2 ± 4.7	51.9 ± 4.8
Consumption of remifentanil (µg)	713.4 ± 65.5	700.6 ± 63.7	688.9 ± 60.2

Note: Data are presented as the mean ± standard deviation (SD) or as the number of the patients, as appropriate

Abbreviations: ASA, American Society of Anesthesiologists; BMI, body mass index; Group R = Group ropivacaine; Group RD1 = Group ropivacaine plus 0.5 µg/kg dexmedetomidine; Group RD2 = Group ropivacaine plus 1.0 µg/kg dexmedetomidine;

The global QoR-15 scores are shown in Table 2. At the first 12 h after surgery, it was observed that the global QoR-15 score was significantly higher in both the RD1 and RD2 groups in comparison with group R (P < 0.05), however, the global QoR-15 scores were similar in the RD2 and RD1 groups (P > 0.05). On postoperative day 1, the QoR-15 scores were higher in group RD2 than in either group R or group RD1 (P < 0.05), but there was no significant difference between the two latter groups (P > 0.05). Moreover, no statistically significant differences were found in the QoR-15 scores among the three groups at 48 and 72 h after the surgery (P > 0.05).

**Table 2:** QoR-15 scores in the three groups

	Group R	Group RD1	Group RD2	P-value
QoR-15 Score				
Pre-operative	145.2 ± 3.5	146.6 ± 5.2	143.3 ± 10.5	0.421
12 h postoperatively	92.5 ± 8.1	99.5 ± 7.9^a^	104.1 ± 6.9^a^	< 0.001
24 h postoperatively	102.6 ± 10.0	106.4 ± 8.8	119.7 ± 11.1^a,b^	< 0.001
48 h postoperatively	115.0 ± 10.0	114.1 ± 10.0	121.3 ± 11.6	0.506
72 h postoperatively	122.0 ± 9.1	121.3 ± 9.7	126.5 ± 10.5	0.338

Note: Data are presented as the mean ± standard deviation (SD).

Abbreviations:QoR-15, 15-item quality of recovery questionnaire

^a^ P < 0.05 versus group R. ^b^P < 0.05 versus group DR1.

The VAS scores are presented in Table 3. At 12 h after surgery, the VAS score was lower in both the RD1 and RD2 groups than in group R (P < 0.05). At 24 h after surgery, the VAS score was lower in group RD2 than in the R and group RD1 groups (P < 0.05), however there was no difference found between the R and RD1 groups (P > 0.05). Furthermore, no statistically significant differences were observed in the VAS score among the three groups at 4 h, 8 h, and 48 h, 72 h after the surgery (P > 0.05).

**Table 3:** Postoperative VAS scores

	Group R	Group RD1	Group RD2	P-value
VAS Scores in rest				
4 h	2(1–3)	2(1–4)	2(2–3)	0.458
8 h	2(1–4)	2(1–4)	3(1–3)	0.521
12 h	5(4–6)	2(1–4)^a^	3(1–3)^a^	0.035
24 h	5(4–6)	4(3–8)	3(3–4)^ab^	0.027
48 h	3(2–3)	4(3–6)	3(2–5)	0.683
72 h	3(2–4)	3(2–5)	2(1–4)	0.310

Notes: Data are presented as median (interquartile range)

Abbreviations: VAS, visual analog scale

^a^P < 0.05 versus group R. ^b^P < 0.05 versus group DR1.

As shown in Table 4, the cumulative flurbiprofen consumption was reduced during the 24 h after the surgery in group RD2 (P < 0.05). The total number of times the PCIA was pressed was significantly lower in the RD1 and RD2 groups than in group R (P < 0.05), moreover, in comparison to the group RD1, the total number of PCIA presses was markedly reduced in the RD2 group (P < 0.05). The incidence of postoperative nausea and vomiting was lower in group RD2 (P < 0.05). There were no significant differences observed in the incidence of bradycardia, hypotension, or dizziness among the three groups. In addition, none of the patients experienced adverse events associated with ESPB.

**Table 4:** Secondary outcomes during the study period

	Group R	Group RD1	Group RD2	P-value
Total press times of PCIA	16.5(11.9–23.3)	12.5(7.3–17.6)^a^	7.9(4.3–12.5)^ab^	< 0.001
Time to first flurbiprofen(h)	8(6.0–9.0)	12.5(9.0-17.3)^a^	20(18.5–22.5)^ab^	< 0.001
Cumulative flurbiprofen consumption(mg)				
0-24 h	16.0(12.0–24.0)	16.0(14.0–16.0)	8.0(0.0–8.0)^ab^	< 0.001
24-48 h	8.0(8.0–16.0)	16.0(8.0–16.0)	8.0(0.0–8.0)	0.367
48-72 h	8.0(0.0–8.0)	8.0(0.0–8.0)	8.0(0.0–8.0)	0.689
PONV	6	2^a^	0^ab^	< 0.001
Bradycardia	0	0	1	0.786

Note: Data are presented as the mean ± standard deviation (SD), median (interquartile range) or as the number of patients, as appropriate

Abbreviations: PONV, postoperative nausea or vomiting

^a^P < 0.05 versus group R. ^b^P < 0.05 versus group DR1.

## Discussion

In this study, we found that 1 µg/kg Dex, rather than 0.5 µg/kg, used as adjunct in combination with ropivacaine were effective in improving the quality of recovery 24 h after surgery. The results showed that 1 µg/kg Dex used as an adjunct in combination with 0.5% ropivacaine 30 ml for ESPB significantly improved the global QoR-15 scores by 13 points on the first day after VATLS and led to a clinically significant enhancement in the quality of the postoperative recovery [14]. This finding is consistent with a previous study by Li-Yun Zhang and colleagues [15], it was found that intravenous infusion of Dex can also improve the QoR scores at 24 h postoperatively in patients receiving robotic-assisted thoracic surgery. In addition, Dex (1 µg/kg) as an adjunct with ropivacaine also provided superior postoperative analgesia, and reduced the postoperative flurbiprofen consumption and PONV without increasing the side effects associated with Dex in the early postoperative period. In summary, These finding suggested that adding of 1 µg/kg dexmedetomidine as an adjunct to 0.5% ropivacaine for ESPB may be an efficient strategy for enhancing early recovery following VATLS. However, we did not find that 1 µg/kg Dex as an adjunct in combination with ropivacaine significantly improved the quality of recovery at 48 and 72 h postoperatively, this may be due to the pharmacokinetic properties of ropivacaine and Dex. Rancourt et al. reported that the duration of sensory blockade with ropivacaine plus 1 µg/kg Dex was 21.5 h in posterior tibial nerve block [16]. Therefore, in the following research, other long-acting local anesthetics can be invistigated to prolong the duration of analgesia and further improve the quality of postoperative recovery.

There was no significant difference in the VAS score between the three groups at 4 and 8 h after surgery in our study, this observation suggested that ropivacaine nerve block can effectively maintain effective analgesia for 4–8 h with or without Dex, which is consistent with the findings reported by Wang Yihan [17]. The differences in VAS scores at 12 and 24 h between the three groups indicated that Dex in combination with ropivacaine can significantly prolong the duration of erector spinal plane block in a dose-dependent fashion. Brummett also reported that the addition of Dex to ropivacaine can effectively increase the duration of sensory blockade in a dose-dependent manner in a rat model [18]. Moreover, They found that blockage of the sciatic nerve with bupivacaine combined with very high dosages of Dex (20–40 mg/kg) did not show any substantial neurotoxicity, axonal or myelin damage 24 h and 14 d after injection [19], therefore, in the following study, maybe we can explore the potential clinical safety of higher dosages of Dex as an adjunct to nerve blocks and whether it can further prolong the duration of ESPB and improve the quality of postoperative recovery.

Consistent with the earlier reports of Xu xia [20] and Marhofer [21], we did not observe Dex-related side effects in this study, this might be associated with the Dex dosage [22, 23] and the exclusion of older individuals [24]. However, several previous studies have reported that the addition of Dex can significantly reduce blood pressure and heart rate [25, 26], thus, it is important in clinical practice to continuously monitor hemodynamic parameters in patients after Dex administration. In addition, we found a lower incidence of postoperative nausea and vomiting in group RD2, which could be related to the use of Dex. Young Song and colleagues have indicated that Dex may directly inhibit PONV by lowering the plasma concentrations of catecholamines [27]. Xu si qi et al. also found that the use of Dex in laparoscopic hysterectomy could reduce PONV through its analgesic and opioid-sparing effects in the patients [28].

There are several limitations associated with our study. First, we applied the ESPB procedure after the induction of general anesthesia to alleviate patient anxiety, the spread of the sensory blockade and possible block failures were not systematically evaluated, although ultrasound technology allows the depiction of anatomical structures and anesthetic drug diffusion in real-time. Second, we only investigated the quality of postoperative recovery in patients between 18 and 65 years of age. However, with the rapid aging of society, the proportion of elderly patients with underlying cardiovascular disease undergoing thoracic surgery has increased significantly, thus, it is also necessary to explore the efficacy and safety of regional anesthesia combined with different dosages of Dex in elderly patients in future studies. Third, we used intravenous sufentanil PCA to manage postoperative analgesia, however, it would be better to administer postoperative pain through Sublingual sufentanil tablet system [29]. Forth, we did not measure the hemodynamic parameters during or after surgery. Although Yu and colleagues demonstrated that as an adjuvant to ropivacaine in ESPB, Dex can be safely used in thoracoscopic surgery [30], it is still necessary to measure the hemodynamic parameters.

In conclusion, 1 µg/kg Dex (but not 0.5 µg/kg Dex) used as an adjunct in combination with ropivacaine showed a clinically significant enhancement in the quality of recovery at 24 h postoperatively. However, it did not significantly improve the quality of recovery at 48 and 72 h after surgery.

## Data Availability

The datasets generated during and/or analyzed during the current study are available from the corresponding author on reasonable request.

## Abbreviations

ESPB: Erector spinae plane block
QoR: Quality of recovery
VATLS: Video-assisted thoracoscopic lobectomy surgery
Dex: Dexmedetomidine
ASA: American society of anesthesiologists
PACU: Post-anesthesia care unit
PCIA: Patient-controlled intravenous anesthesia
PONV: Postoperative nausea and vomiting
POD: Postoperative days
BIS: Bispectral index
